# O-GlcNAcylation of the intellectual disability protein DDX3X exerts proteostatic cell cycle control

**DOI:** 10.1098/rsob.250064

**Published:** 2025-07-02

**Authors:** Conor W. Mitchell, Huijie Yuan, Marie Sønderstrup-Jensen, Andrew T. Ferenbach, Daan M. F. van Aalten

**Affiliations:** ^1^ Department of Molecular Biology and Genetics, Aarhus Universitet, Aarhus, Denmark; ^2^ Division of Molecular, Cell and Developmental Biology, School of Life Sciences, University of Dundee, Dundee, UK, Dundee, UK

**Keywords:** O-GlcNAc, cell cycle, cell signalling

## Abstract

O-GlcNAcylation of intracellular proteins is a key regulator of diverse cellular and developmental processes. Previous studies have demonstrated the acute sensitivity of cell cycle progression to chemical and genetic manipulation of O-GlcNAc homeostasis. However, the mechanisms by which O-GlcNAc regulates the cell cycle remain poorly understood. Here, we report Ser584 O-GlcNAcylation of the RNA helicase DDX3X, a microcephaly associated protein, as a proteostatic mechanism regulating S-phase entry. Loss of Ser584 O-GlcNAcylation promoted degradation of DDX3X by the proteasome, resulting in reduced expression of the DDX3X target gene *cyclin E1* and impaired cell cycle progression from G1 to S phase. These findings display how a single O-GlcNAc site affects DDX3X stability and thereby the cell cycle.

**Figure:**
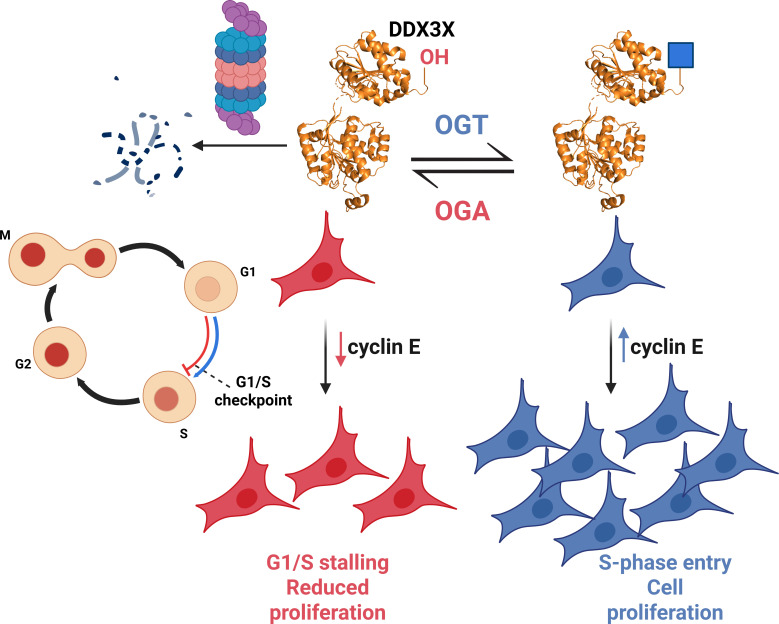

## 1. Introduction

O-GlcNAcylation, the attachment of a single monosaccharide unit of β-N-acetyl-D-glucosamine to protein Ser/Thr residues, is a dynamic post-translational modification (PTM) occurring on >9000 nuclear, cytoplasmic and mitochondrial proteins [1,2]. Since its discovery in 1984, O-GlcNAcylation has emerged as a critical regulator of diverse developmental and cellular processes, including transcription/translation [3], differentiation [4], neurite outgrowth [5] and cell cycle progression, among others [6–8]. The ability of O-GlcNAc to modulate these processes stems, in part, from the dynamic nature of this PTM, with the sole O-GlcNAc transferase (OGT) catalysing O-GlcNAc transfer and the antagonistic O-GlcNAc hydrolase (OGA) removing O-GlcNAc multiple times during the lifespan of a protein substrate in response to extra- and intra-cellular cues [9,10]. Mutations in either OGT or OGA segregate with neurodevelopmental disorders [11–15], and knock-out of the *Ogt* gene is embryonic lethal in mice [16], underscoring the essentiality of O-GlcNAcylation in eukaryotic biology.

Cell cycle progression is a critical process for the maintenance and propagation of eukaryotic life and is dependent on the timely expression and activation of structural proteins, kinases and transcription factors to ensure faithful replication and transmission of the parent cell genome to segregated daughter cells. In particular, the canonical cyclin/cyclin-dependent kinase (CDK) family is essential for activation and commitment of cells to progression through the cell cycle [17], with mutations in CDK family members and elevated cyclin expression segregating with disorders including cancer and microcephaly [18,19]. Previous studies have broadly highlighted the importance of O-GlcNAc homeostasis in CDK/cyclin regulation and cell cycle progression through over-expression and chemical inhibition of the O-GlcNAc cycling enzymes [6,20]. OGT over-expression results in dysregulated cyclin expression and genomic instability due to increased aneuploidy [6]. Chemical inhibition of OGA similarly affects cyclin expression patterns with failure to induce cyclin E expression during G1 and stalled G1/S phase progression [6]. Although the broad morphological and cytological effects of disrupted O-GlcNAc homeostasis on cell cycling have been reported, the O-GlcNAc signalling nodes responsible for up/downstream regulation of cell cycle progression remain largely unknown, with limited mechanistic dissection reported to date.

Previous work has identified several O-GlcNAc-regulatory mechanisms of the cell cycle across the earlier G0/G1 phases and the later M phase. For example, O-GlcNAcylation stabilizes β-catenin and may potentiate β-catenin-dependent *cyclin D* transcription to promote cell cycle re-entry from G0 [21,22]. Additionally, in an unusual example of OGT’s functional diversity, Host Cell Factor 1 (HCF-1) is cleaved at one of six proteolysis repeats by OGT in the same active site that is required for catalysing O-GlcNAc transfer [23,24]. The N- and C-terminal proteolysis products of HCF-1 cleavage, HCF1_N_ and HCF1_C_, subsequently play separate roles in G1 and M phase progression, respectively [25]. Curiously, HCF-1 is heavily O-GlcNAcylated at over 30 modification sites [26], and loss of O-GlcNAcylation on HCF-1 correlates with binucleation defects in carcinoma cells, a marker of failed cytokinesis or cleavage furrow regression, suggesting distinct roles for HCF-1 cleavage and O-GlcNAcylation in cell cycle progression and mitosis [23]. Regarding M phase, OGT over-expression results in reduced transcription of the CDK1 activating phosphatase CDC25c and downstream hypo-phosphorylation of CDK1 substrates implicated in mitotic spindle formation [20]. To summarize, aside from these examples and several others [27,28], detailed mechanistic explanations for how O-GlcNAcylation regulates cell cycle progression, a critical aspect of eukaryotic biology, are largely lacking.

Here, we dissect the interplay between O-GlcNAcylation and the cell cycle through a combination of bioinformatic and cellular approaches to identify novel regulators of the cell cycle. Through bioinformatic analysis of *OGT* and *OGA* correlated genes, we find evidence for extensive, regulatory functions of O-GlcNAcylation in the cell cycle. Focusing on the product of one of the strongest *OGT*/*OGA* correlated genes, the RNA helicase DDX3X, we report O-GlcNAcylation of a single site on DDX3X, Ser584, as a proteostatic regulator of cell cycle progression. DDX3X unwinds complex secondary structures in the 5′ untranslated region (UTR) of *cyclin E* mRNA, thereby promoting efficient scanning for AUG start codons by the pre-initiation complex [29,30]. Cyclin E is critical for commitment of cells to S phase entry late in G1 through activation of CDK2 and hyper-phosphorylation of the tumour suppressor, retinoblastoma (Rb) [31]. A previous bioinformatic study of the O-GlcNAcome highlighted the presence of two O-GlcNAc sites—Ser584 and Ser588—in the DDX3X C-terminal extension (CTE), deletions of which resulted in microcephaly in patients [32,33]. We report here that Ser584 O-GlcNAcylation prevents proteasomal degradation of DDX3X, promoting *cyclin E* translation and passage from G1 into S-phase.

## 2. Results

### 2.1. Expression of cell cycle regulators, including *DDX3X*, correlates with *OGT* and *OGA*

**Figure 1.**
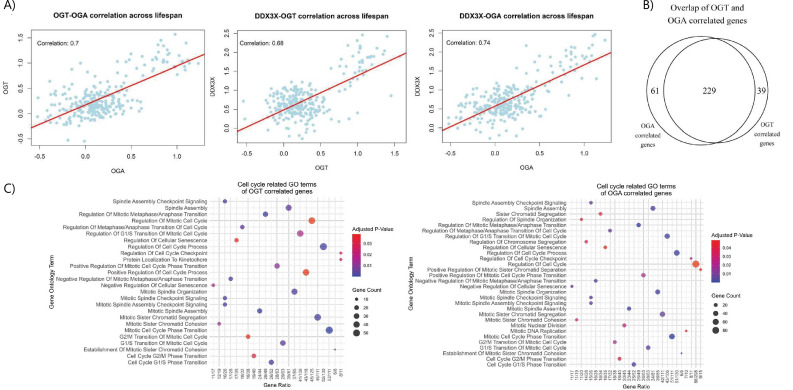
Co-expression analysis of *OGT* and *OGA* in the prefrontal cortex identifies correlated cell cycle-associated genes and processes. (A) Scatterplots showing correlation between *OGT* and *OGA* (*r* = 0.7 and *p* < 0.05) and *DDX3X* and *OGT* (*r* = 0.68 and *p* < 0.05) and *OGA* (*r* = 0.74 and *p* < 0.05), respectively.(B) Venn diagram showing the number of genes that overlap between the genes involved in the cell cycle-related GO BP terms (adj. *p* < 0.05) and correlated with *OGT* and *OGA* (*r* > 0.4 and *p* < 0.05), respectively. Co-expression analysis was performed on the 17 160 genes from the BrainCloud transcriptomics dataset (*n* = 267) (for details see §4). The number of cell cycle-related genes correlated (*r* > 0.4) and anticorrelated (*r* < −0.4) with *OG*T and *OGA*, respectively, are annotated (*p* < 0.05). (C) Bubble charts showing the enriched GO BP terms related to cell cycle for both *OGT* and *OGA* correlated genes, respectively (adj. *p* < 0.05).

As an unbiased strategy towards identifying signalling nodes and pathways regulated by OGT/OGA in the cell cycle, we analysed the BrainCloud transcriptomic dataset (that reports the prefrontal cortical transcriptome across the human lifespan) for *OGT*/*OGA* correlated genes [34]. Across over 17 000 genes, we observed 4273 genes correlated with *OGT* (Pearson correlation coefficient (*r*) >0.5; Student’s asymptotic test, *p* < 0.05; electronic supplementary material, S1), and 4436 genes correlated with *OGA* throughout the human lifespan (electronic supplementary material, S1). As expected, a strong correlation was observed between *OGT* and *OGA* (*r* = 0.7), consistent with previous reports highlighting the tight coupling of *OGT* and *OGA* transcription to maintain O-GlcNAc homeostasis (figure 1A) [35,36]. Gene ontology (GO) analysis for biological processes (BPs) of genes correlated with *OGT* identified several clusters of related biological processes, including chromatin remodelling, DNA damage response, and the stress response, all previously reported to be regulated by O-GlcNAcylation (see electronic supplementary material, S2 for the full list of enriched GO terms) [8,27,37]. Interestingly, we observed GO terms related to the cell cycle as among the most enriched *OGT*-correlated processes (26 cell cycle-related GO terms out of 155 enriched GO terms for *OGT*-correlated genes), with 6% of all *OGT*-correlated genes (268) annotated as being related to cell cycle progression (see electronic supplementary material, S2 for the full list of GO terms). Such genes included the M-phase regulator CDK1 and its upstream inhibitory kinase Wee1, as well as the S-phase regulatory kinases CDK4, CDK6 and CDK2. *OGA* correlation analysis revealed a similar observation, with 290 cell-cycle-related genes correlating with *OGA*, representing 7% of all *OGA* correlated genes across 160 cell cycle GO terms (electronic supplementary material, S1; figure 1C), including the tumour suppressor RB1 and the G1/S phase repressor CDK7. Furthermore, there was extensive overlap between the lists of *OGT* and *OGA* correlated cell cycle genes, with 229 correlating with both O-GlcNAc cycling enzymes (figure 1B), perhaps expected given the dynamic and antagonistic nature of these enzymes in regulating O-GlcNAc signalling. Additionally, consistent with the overlapping nature of the *OGT* and *OGA* correlated gene lists, we observed functionally similar enriched GO terms between the *OGT* and *OGA* correlated gene sets, including genes related to double-stranded break repair, histone H4 acetylation and the stress response (electronic supplementary material, S2).

Amongst the *OGT* and *OGA* correlated genes implicated in cell cycle regulation, the gene encoding the RNA helicase *DDX3X*, an RNA helicase linked to S-phase entry and intellectual disability, was among the strongest *OGT/OGA* correlated genes (*r* = 0.68 and *r* = 0.74, respectively; figure 1A) [29,33,38]. Interestingly, DDX3X is itself an O-GlcNAc protein, with a previous O-GlcNAc proteomic study identifying two O-GlcNAc sites—Ser584 and Ser588—in the CTE of DDX3X [26]. In parallel, we noted that deletion of the CTE segregated with microcephaly and intellectual disability [33], and truncation of the CTE was reported to attenuate DDX3X RNA helicase activity in a separate study [39]. These convergent lines of evidence supported a possible regulatory function for O-GlcNAc in regulating DDX3X activity and, potentially, S-phase entry during the cell cycle. We therefore focused our efforts on dissecting the function of O-GlcNAc on DDX3X.

### 2.2. Global manipulation of O-GlcNAc homeostasis affects DDX3X protein levels

**Figure 2.**
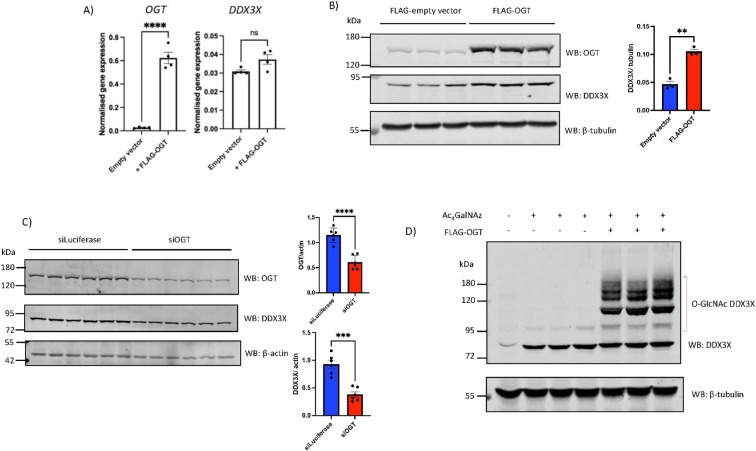
Manipulating global O-GlcNAc homeostasis elevates DDX3X protein levels and O-GlcNAcylation without altering DDX3X transcription. (A) *OGT* and *DDX3X* mRNA levels after transfection of HEK293T cells with either a FLAG-empty CMV vector or equal quantity of FLAG-OGT^WT^ plasmid. *OGT* and *DDX3X* mRNA levels were normalized to the β-actin housekeeping gene. *n* = 4 technical replicates, plotted as the mean ± s.e.m. Data were analysed using Student’s unpaired *t*‐test. ns: not significant, ****p < 0.0001. (B) Endogenous DDX3X protein levels after transfection of HEK293T cells in triplicate with either FLAG-empty vector or FLAG-OGT^WT^ plasmid. OGT levels are reported as a control for successful transfection with FLAG-OGT^WT^. *n* = 3 technical replicates. Endogenous DDX3X levels, normalized to β-tubulin, are plotted ±s.e.m. Data were analysed using Student’s unpaired *t*‐test. **p < 0.01. (C) Endogenous DDX3X and OGT protein levels after transfection with siRNA targeting firefly luciferase (non-targeting control) or siRNA targeting human OGT. DDX3X and OGT levels, normalized to β-actin, are plotted ±s.e.m. *n* = 6 technical replicates. Data were analysed using Student’s unpaired *t*‐test. ***p < 0.001, ****p < 0.0001. (D) HEK293T cells were transfected with either FLAG-empty vector or plasmid encoding FLAG-OGT^WT^ for 48 h. 16 h prior to cell lysis, cells were treated with 200 μM Ac_4_GalNAz to label O-GlcNAc sites with GlcNAz (see §2 for description of approach) or an equal volume of DMSO (vehicle only control). GlcNAz-labelled proteins were conjugated to PEG 5 kDa mass tags via strain-promoted azide alkyne cycloaddition, and reaction products analysed via DDX3X Western blot. The shifted bands within the bracket represent PEGylated DDX3X that was labelled with GlcNAz.

To investigate whether the observed correlation between *OGT* and *DDX3X* translates to the protein level, and thus whether regulatory interplay exists between these two enzymes, OGT was transiently over-expressed in HEK293T cells and DDX3X protein and mRNA levels were measured. Global elevation of O-GlcNAc levels via OGT over-expression in HEK293T cells increased endogenous DDX3X protein levels without increasing DDX3X mRNA levels (figure 2A,B), whereas over-expression of a catalytically null OGT^K852M^ mutant had no effect on DDX3X protein levels (electronic supplementary material, figure S1). Conversely, knock-down of OGT using small interfering RNA (siRNA) resulted in reduced DDX3X protein levels (figure 2C). The accumulation of DDX3X protein, but not mRNA, following OGT over-expression suggested that elevated DDX3X protein levels resulted from either a post-transcriptional or post-translational effect of elevated O-GlcNAcylation on DDX3X levels. To probe this further, a metabolic labelling strategy was used to measure DDX3X O-GlcNAcylation stoichiometry in the presence or absence of exogenous OGT. Towards this end, HEK293T cells were treated with tetra-acetylated GalNAc azide (Ac_4_GalNAz). Unlike Ac_4_GlcNAz, this cell-penetrant metabolic precursor is efficiently converted into UDP-GalNAz via the hexosamine salvage pathway, and subsequently epimerized to UDP-GlcNAz [40]. Importantly, the azide group is tolerated by OGT, allowing UDP-GlcNAz to be used as a donor substrate for labelling endogenous GlcNAc sites with GlcNAz. After metabolic labelling, endogenous GlcNAzylated proteins were conjugated to resolvable PEG mass tags via strain-promoted azide alkyne cycloaddition (SPAAC). In the absence of OGT, DDX3X shows a single resolvable band shift, compatible with mono-GlcNAcylation of the protein (figure 2D), whereas over-expression of OGT resulted in accumulation of poly-GlcNAcylated glycoforms of DDX3X (figure 2D). Taken together, these data demonstrate that manipulating O-GlcNAc homeostasis affects DDX3X protein levels, suggesting O-GlcNAc-dependent regulation of DDX3X.

### 2.3. Ser584 O-GlcNAcylation affects DDX3X levels

**Figure 3.**
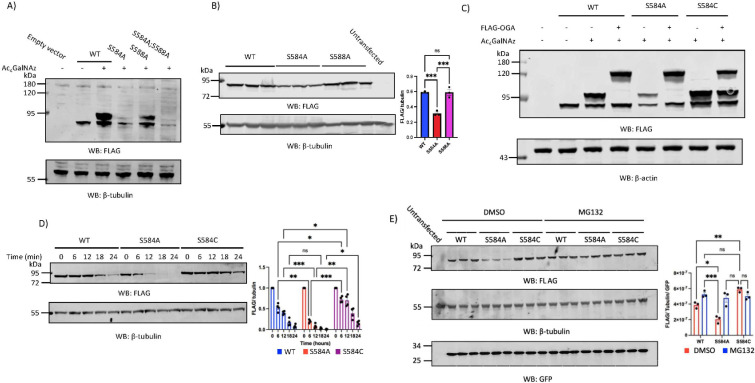
Ser584 O-GlcNAcylation protects DDX3X from degradation by the ubiquitin-proteasome system. (A) HEK293T cells were transfected with FLAG-empty vector, N-terminally FLAG-tagged DDX3X WT, S584A, S588A, or a S584A;S588A double alanine mutant, followed by metabolic labelling of O-GlcNAc sites with 200 μM Ac_4_GalNAz. As a negative control, a FLAG-DDX3X^WT^ transfected well was treated with an equal volume of DMSO (vehicle only control) instead of Ac_4_GalNAz. After transfection and metabolic labelling, GlcNAz-labelled proteins were conjugated to 5 kDa PEG mass tags via strain-promoted azide-alkyne cycloaddition, and FLAG-DDX3X O-GlcNAcylation stoichiometry analysed by blotting for the N-terminal FLAG tag. (B) HEK293T cells were transfected in triplicate with FLAG-DDX3X^WT^, FLAG-DDX3X^S584A^ or FLAG-DDX3X^S588A^. Cells treated only with equivalent volumes of lipofectamine without plasmid were used as a negative control. FLAG-tagged DDX3X levels were measured via FLAG-tag Western blot and normalized to β-tubulin. Normalized FLAG-DDX3X levels are plotted as the mean ±s.e.m. Data were analysed using one-way ANOVA (*n* = 3 technical replicates). ns: not significant; ***p < 0.001. (C) HEK293T cells were either transfected with one of FLAG-DDX3X^WT^, FLAG-DDX3X^S584A^ or FLAG-DDX3X^S584C^ only, or additionally co-transfected with FLAG-OGA^WT^, for 48 h. 16 h prior to cell lysis, transfected cells were treated with either 200 μM Ac_4_GalNAz or an equal volume of DMSO (vehicle only control). After co-transfection and metabolic labelling, GlcNAz-labelled sites were conjugated to 5 kDa PEG mass tags via strain-promoted azide alkyne cycloaddition, and DDX3X O-GlcNAcylation stoichiometry measured via FLAG-tag Western blot. As a control for the specificity of the FLAG antibody, cells were also transfected with a FLAG-empty vector. The FLAG signal at ~120 kDa corresponds to the expected molecular weight of transfected FLAG-OGA^WT^. (D) HEK293T cells were transfected with either FLAG-DDX3X^WT^, FLAG-DDX3X^S584A^ or FLAG-DDX3X^S584C^ prior to addition of 100 μg ml^−1^ cycloheximide for 0,6,12,18 or 24 h. The turnover of each FLAG-DDX3X construct was measured by normalizing the FLAG signal at each time-point to both β-tubulin and the FLAG signal at T = 0. Data are plotted as the mean ±s.e.m. Data were analysed using two-way ANOVA (*n* = 3 technical replicates). ns: not significant; *p < 0.05; **p < 0.01; ***p < 0.001. (E) HEK293T cells were transfected with either a FLAG-empty vector or a dual expression vector encoding FLAG-DDX3X^WT^, FLAG-DDX3X^S584A^ or FLAG-DDX3X^S584C^, and a 3’ P2A sequence that is co-translationally cleaved followed by the open reading frame for GFP (see §2 for a description of the P2A-GFP vector). Transfected cells were treated with either 10 μM MG132 or an equal volume of DMSO for 16 h prior to cell lysis. Transfected DDX3X levels were quantified via normalization of the FLAG-tag signal to both the β-tubulin housekeeping protein and the co-expressed GFP reporter. Normalized FLAG-DDX3X levels are plotted as the mean ±s.e.m. Data were analysed using a one-way ANOVA (*n* = 3 technical replicates). ns: not significant, **p < 0.05, **p < 0.01, ***p < 0.001.

The observed accumulation of O-GlcNAcylated DDX3X following OGT over-expression suggested that O-GlcNAcylation of DDX3X results in elevated DDX3X protein levels, though the mechanism underpinning this remained unclear. To interrogate the possible site-specific effects of DDX3X O-GlcNAcylation, we mapped DDX3X O-GlcNAc sites using the metabolic labelling/PEG mass tagging strategy described above. A previous O-GlcNAcomics study in T-lymphocytes mapped two sites of DDX3X O-GlcNAcylation at Ser584 and Ser588 in the CTE [26]. However, the PEG mass tagging strategy used here suggests a single site of modification (figure 2D). To determine which of these sites makes significant contributions to DDX3X O-GlcNAcylation stoichiometry, HEK293T cells were transiently transfected with FLAG-tagged DDX3X WT, Ser584Ala, Ser588Ala and Ser584Ala; Ser588Ala double alanine mutants, and O-GlcNAc sites were labelled with DBCO-mPEG as described above. Of the single alanine mutants analysed, FLAG-DDX3X^Ser584Ala^ showed the greatest decrease in O-GlcNAcylation stoichiometry, with the intensity of the single band shift observed for FLAG-DDX3X^WT^ reduced by 60% for FLAG-DDX3X^Ser584Ala^ (figure 3A, lane 4). Curiously, FLAG-DDX3X^Ser588Ala^ also showed 20% reduced O-GlcNAcylation stoichiometry (figure 3A, lane 5), and >90% abolition of the shifted band was only observed for the double alanine FLAG-DDX3X^Ser584Ala;Ser588Ala^ mutant (figure 3A, lane 6). We cannot discard the possibility that mutagenesis of Ser584 to Ala affects OGT activity on Ser588 and vice versa, resulting in artefactual gain or loss of OGT activity towards the remaining O-GlcNAc site. Moreover, the residual O-GlcNAcylation of FLAG-DDX3X^Ser584Ala;Ser588Ala^ suggests that additional lower occupancy O-GlcNAc sites may be present on DDX3X, an assertion supported by the 4 O-GlcNAc sites observed on endogenous DDX3X following OGT over-expression (figure 2D). In addition to providing information on O-GlcNAc site localization and stoichiometry, the single and double alanine mutants also suggested an effect on the steady-state levels of DDX3X in cells, as the Ser584Ala single and Ser584Ala; Ser588Ala double alanine mutants both expressed at lower levels than WT (figure 3A). Indeed, repetition of transfection experiments in triplicate showed a 60% reduction in steady-state expression of FLAG-DDX3X^Ser584Ala^ and FLAG-DDX3X^Ser584Ala;Ser588Ala^ but not FLAG-DDX3X^Ser588Ala^ (figure 3B). This 60% decrease in FLAG-DDX3X^Ser584Ala^ protein level was proportional to the decrease in DDX3X O-GlcNAcylation stoichiometry after alanine mutagenesis of Ser584 (figure 3A). Melting curves of DDX3X^WT^ and DDX3X^Ser584Ala^ recombinant protein, expressed in *Escherichia coli*, did not differ (electronic supplementary material, figure S2), indicating that the differences in FLAG-DDX3X^WT^ and FLAG-DDX3X^Ser584Ala^ expression levels did not stem from the alanine mutation disrupting the protein fold. As an additional control, transfection of HEK293T cells with FLAG-DDX3X plasmids where the 3’ end of the DDX3X ORF was fused via a co-translationally cleaved P2A linker to GFP, revealed no differences in GFP protein levels (figure 3E), suggesting that the observed reduction in steady-state FLAG-DDX3X^Ser584Ala^ levels did not stem from differential transfection or translation efficiencies and therefore results from loss of O-GlcNAcylation. To summarize, Ser584 O-GlcNAcylation affects DDX3X protein levels.

### 2.4. Ser584 O-GlcNAcylation prevents DDX3X degradation by the ubiquitin-proteasome system

The effects of protein O-GlcNAcylation on proteostasis have been well documented, with examples of O-GlcNAcylation inhibiting protein aggregation, increasing protein thermostability, and inhibiting degradation via the ubiquitin-proteasome system (UPS), reported previously [41–44]. The observation that over-expression of OGT elevated DDX3X O-GlcNAcylation and protein levels without affecting DDX3X transcription, paired with observed reductions in the levels of the Ser584Ala mutant, suggested a post-translational mechanism through which O-GlcNAc stabilizes DDX3X. To investigate this hypothesis, FLAG-DDX3X^WT^ and FLAG-DDX3X^Ser584Ala^ were transiently over-expressed, and their turnover rates *in cellulo* analysed after blocking translation with the protein synthesis inhibitor cycloheximide. The rate of FLAG-DDX3X^Ser584Ala^ turnover was 3-fold higher than for FLAG-DDX3X^WT^ (figure 3D), with no detectable FLAG-DDX3X^Ser584Ala^ after 18 h cycloheximide treatment, in contrast to FLAG-DDX3X^WT^ where 20% of the initial pool of FLAG-DDX3X^WT^ could be detected at the same time point (figure 3D). Moreover, treatment of FLAG-DDX3X-transfected cells with the proteasome inhibitor MG132 rescued FLAG-DDX3X^Ser584Ala^ expression (figure 3E), suggesting the increased turnover of FLAG-DDX3X^Ser584Ala^ stemmed from increased degradation via the UPS. As an additional control, we installed an OGA-resistant S-GlcNAc analogue of O-GlcNAc at Ser584 via mutation of this residue to Cys. This approach exploits a recently discovered OGT promiscuity towards Cys, combined with the observation that S-GlcNAcylated Cys are resistant to OGA hydrolysis, thus resulting in site-specific elevation/installation of GlcNAcylation in the context of an otherwise unaltered O-GlcNAcome [45]. FLAG-DDX3X^S584C^ demonstrated a 30% increase in O-GlcNAcylation stoichiometry relative to FLAG-DDX3X^WT^, and while co-transfection with FLAG-OGA^WT^ abolished O-GlcNAcylation of FLAG-DDX3X^WT^ and FLAG-DDX3X^S584A^, the intensity of the shifted band was only reduced by 20% for FLAG-DDX3X^S584C^ (figure 3C). The 20% reduction in FLAG-DDX3X^S584C^ O-GlcNAcylation stoichiometry after OGA over-expression could reflect removal of Ser588 O-GlcNAcylation with preservation of S-GlcNAc at Cys584. Hyper-S-GlcNAcylated FLAG-DDX3X^Ser584Cys^ showed higher steady-state expression levels than FLAG-DDX3X^WT^ (figure 3E), which could not be elevated further by the addition of MG132. Hyper-S-GlcNAcylated FLAG-DDX3X^Ser584Cys^ also showed a reduced turnover rate in the cycloheximide assay compared to FLAG-DDX3X^WT^ (figure 3D). Taken together, these data suggest that Ser584 O-GlcNAcylation protects DDX3X from degradation by the UPS.

### 2.5. Loss of DDX3X O-GlcNAcylation impairs cellular proliferation and stalls entry into S-phase in a cyclin E1-dependent manner

**Figure 4.**
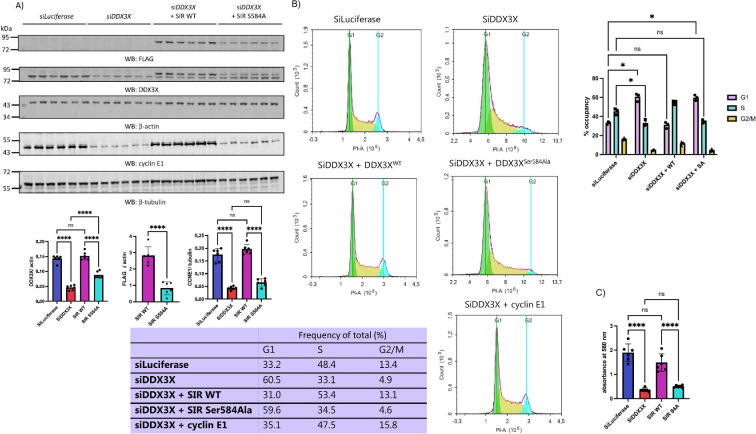
Loss of DDX3X Ser584 O-GlcNAcylation impairs S-phase entry and stalls cellular proliferation. (A) HEK293T cells were transfected with siRNA against either firefly luciferase (non-targeting control) or endogenous human DDX3X. In parallel, DDX3X knock-down cells were also co-transfected with either siRNA-resistant (SIR) FLAG-DDX3X^WT^ or SIR FLAG-DDX3X^S584A^. After 72 h, live cell numbers were counted (see electronic supplementary material, figure S3), and cells lysed for analysis of FLAG-DDX3X, total (endogenous and FLAG-DDX3X) DDX3X, and cyclin E1 levels by Western blotting. The FLAG, total DDX3X, and cyclin E1 signals, normalized to either β-actin (FLAG, total DDX3X) or β-tubulin (cyclin E1), are plotted as the mean ±s.e.m. Normalized total DDX3X and cyclin E1 data were analysed by one-way ANOVA (*n* = 6 technical replicates). ns: not significant; ****p < 0.0001. Normalized FLAG-DDX3X data were analysed using Student’s unpaired *t*‐test (*n* = 6 technical replicates). ****p < 0.0001. Data presented are from one of two independent experiments. (B) Cell cycle distribution of HEK293T cells transfected with siLuciferase or siDDX3X, or co-transfected with siDDX3X and either SIR FLAG-DDX3X^WT^ or FLAG-DDX3X^S584A^ as determined by flow cytometry. Cells in G1, S, and G2/M are highlighted in green, yellow and cyan, respectively. The average occupancy of each cell population in G1,S and G2/M is listed in table format. Data from three independent experiments were analysed using two-way ANOVA. ns: not significant; *p < 0.05. (C) 1 × 10^5^ cells were transfected with siRNA against luciferase or DDX3X, with siDDX3X cells additionally co-transfected with SIR FLAG-DDX3X^WT^ or SIR FLAG-DDX3X^S584A^. After 72 h, media was aspirated and replaced with 1 ml of 0.5 mg ml^−1^ thiazolyl blue tetrazolium bromide dissolved in cell media for 4 h. After 4 h, DMSO was added to solubilize formazan crystals, and the absorbance at 560 nm was measured. Absorbance measurements are plotted as the mean ±s.e.m. *n* = 6 technical replicates. Data were analysed using one-way ANOVA. ns: not significant; ****p < 0.0001.

DDX3X is a multi-functional RNA helicase, with RNA helicase-dependent and independent roles in cellular proliferation [29,46], the stress response [47] and neurite outgrowth [48]. A previous study in immortalized cell lines linked DDX3X to cell cycle progression through the ability of DDX3X to promote *cyclin E1* translation, thus permitting entry into S phase of the cell cycle [29]. Given that loss of Ser584 O-GlcNAcylation results in increased turnover of DDX3X, we hypothesized that loss of DDX3X O-GlcNAcylation and consequential loss of DDX3X protein may result in reduced cyclin E1 expression. In such a situation, the reduced pool of cyclin E1 would be expected to stall passage from G1 into S phase, and thus reduce the rate of cellular proliferation. As a preliminary investigation, endogenous DDX3X was knocked-down, and knock-down (KD) cells were co-transfected with either siRNA-resistant (from here-on, SIR) FLAG-DDX3X^WT^ or FLAG-DDX3X^Ser584A^. Transfection of these constructs was titrated to rescue total DDX3X levels to those observed in cells transfected with siRNA targeting firefly luciferase (non-targeting control) to prevent artefactual results due to supra-physiological levels of DDX3X. After 72 h of transfection, DDX3X KD reduced live cell counts by 79% and 84% relative to the non-targeting control as measured by trypan blue staining and MTT assays, respectively (figure 4C; electronic supplementary material, figure S3). Co-transfection with SIR FLAG-DDX3X^WT^ rescued this reduction in viability, whereas SIR FLAG-DDX3X^Ser584Ala^ did not (figure 4C; electronic supplementary material S3). Western blotting of cell lysates revealed the same effect of loss of Ser584 O-GlcNAcylation as observed in previous experiments (figure 4A), with KD reducing DDX3X protein levels by 61%, and SIR FLAG-DDX3X^WT^ but not SIR FLAG-DDX3X^Ser584Ala^ rescuing total DDX3X levels (figure 4A). Levels of cyclin E1, the translation of which requires DDX3X activity, were reduced by 71% following DDX3X KD, and this was rescued by SIR FLAG-DDX3X^WT^ but not SIR FLAG-DDX3X^Ser584Ala^, suggesting an S-phase entry defect upon loss of DDX3X Ser584 O-GlcNAcylation (figure 4A).

Although our siRNA-rescue data pointed to a cell proliferation defect upon loss of DDX3X Ser584 O-GlcNAcylation, we were interested in determining whether loss of DDX3X also resulted in increased apoptosis or necroptosis. We therefore knocked-down *DDX3X* and visualized the population of apoptotic and necroptotic cells by staining with both annexin V antibody and propidium iodide (PI). Cells positive for annexin V and PI staining are necroptotic, whereas cells positive for annexin V only are apoptotic [49,50]. Treatment with 2 μM staurosporine, a bacterial alkaloid known to induce apoptosis, as a positive control resulted in 8.5-fold and 12-fold increases in the percent of apoptotic and necroptotic cells, respectively (electronic supplementary material, figure S4, cf. top left-hand panel and top right-hand panel). Knock-down of *DDX3X*, however, did not result in any increase in the apoptotic or necroptotic cell populations compared to the siLuciferase (non-targeting) control (electronic supplementary material, figure S4). We therefore concluded that loss of DDX3X protein does not induce apoptosis or necroptosis in this cell line.

Loss of DDX3X activity has previously been reported to reduce translation of *cyclin E1*, resulting in a stalled S-phase entry defect. Given that loss of Ser584 O-GlcNAcylation correlated with reduced cell proliferation and loss of cyclin E1 protein, we hypothesized that cells expressing FLAG-DDX3X^Ser584Ala^ accumulated in G1 due to a failure to enter S-phase. After repeating our knock-down/rescue experiments and analysing cell cycle progression by flow cytometry, we observed a twofold increase in the percentage of cells in G1 and a 30% reduction in the S-phase population following DDX3X knock-down (figure 4B). Whereas co-transfection of SIR FLAG-DDX3X^WT^ with DDX3X siRNA rescued this defect, co-transection with FLAG-DDX3X^Ser584Ala^ did not. Indeed, the percentage of cells in G1, S and G2/M differed by less than 10% between DDX3X knock-down and SIR FLAG-DDX3X^Ser584Ala^ cells (figure 4B), indicating that loss of Ser584 O-GlcNAcylation reduces the pool of active DDX3X to a level below what is required for efficient *cyclin E1* translation and S-phase entry. To validate the assertion that the observed accumulation of cells in G1, and their failure to enter S-phase, stemmed from impaired cyclin E1 translation, we transfected DDX3X knock-down cells with a cyclin E1 over-expression plasmid. Cyclin E1 transfection rescued the G1-accumulation defect, with the percentage of cells in G1, S and G2/M rescued to the levels observed in the siLuciferase (non-targeting) control (figure 4B). Taken together, loss of Ser584 O-GlcNAcylation decreases levels of DDX3X through increased turnover sufficiently to reduce cyclin E1 levels, resulting in stalled cell cycle progression from G1 to S phase. Thus, DDX3X Ser584 O-GlcNAcylation appears to act as a proteostatic regulator of cell cycle progression.

## 3. Discussion

Since its discovery in 1984, O-GlcNAcylation has emerged as a modulator of diverse cellular and developmental processes. As enrichment and detection methodologies for identifying and mapping O-GlcNAc sites on proteins have improved, the size of the O-GlcNAc proteome has grown substantially over the past decade to include over 9000 intracellular proteins implicated in a wide range of cellular processes [2]. In particular, O-GlcNAc has been identified on regulators of the cell cycle and mitosis [2,26,51], although the site-specific roles of O-GlcNAc remain largely unknown.

As a starting point towards identifying possible O-GlcNAc-regulated pathways in the cell cycle, we analysed transcriptomic data from the human prefrontal cortex to identify genes and biological processes that correlated with the O-GlcNAc cycling enzymes, rationalizing that such correlations may reflect regulatory interplay between O-GlcNAc and key cellular processes, including the cell cycle. For instance, we observed that expression of the master M-phase regulator CDK1 and its upstream regulatory phosphatase CDC25 were correlated with OGT in the human prefrontal cortex. CDC25 activates CDK1 by dephosphorylation of Ser14 and Tyr15 on CDK1 to promote M-phase progression [52], and the observation that both CDK1 and CDC25 positively correlate with OGT expression suggests a layer of O-GlcNAc-dependent transcriptional regulation in promoting M-phase progression. Indeed, a previous study reported delayed M-phase progression following OGA over-expression [6]. Future work focused on investigating the putative regulatory effects of O-GlcNAcylation on CDK1 transcription and its regulation by CDC25 may shed light on the poorly understood role of O-GlcNAc in M-phase progression. Aside from M-phase, we observed positive correlations between the master G1/S-phase regulators CDK4, CDK6 and CDK2 with OGT. The CDK4/6-cyclin D and CDK2-cyclin E complexes function maximally in early and late G1 phase, respectively, and are required for hyperphosphorylation of retinoblastoma (Rb), amongst other targets [53]. Rb hyperphosphorylation liberates the transcription factor E2F from a repressive Rb-E2F complex enabling transcription of genes required for DNA replication [54]. Previous work has pointed to a convergence between O-GlcNAc and the G1/S transition of the cell cycle, with OGA over-expression reducing Rb phosphorylation and uncoupling cyclin E1 expression from G1 phase progression [6]. Additionally, global depression of O-GlcNAc through OGT inhibition and OGA over-expression both result in delayed S-phase entry [28]. Although these studies suggest an important role for O-GlcNAc in S-phase entry, it remains unclear how individual O-GlcNAcylation events regulate S-phase entry, especially at the level of site-specific effects.

The transcriptomic analysis described here identified the cell cycle regulator DDX3X as one of the strongest OGT-correlated genes. DDX3X is an RNA helicase of the DEAD box family that regulates cell cycle progression at both the translational and transcriptional levels and is critical for translation of cyclin E1 and co-regulating transcription of the CDK inhibitor p21 [29,55]. Indeed, loss of DDX3X results in either G1/S phase stalling or global delays in cell cycle progression depending on the cell type studied [29,46,56]. Given the correlation between DDX3X and OGT, and our interest in identifying novel cell cycle regulators, we focused on investigating the possible regulatory interplay between O-GlcNAc and DDX3X. We have shown that Ser584 O-GlcNAcylation acts as a proteostatic regulator of DDX3X activity, with loss of Ser584 O-GlcNAcylation inhibiting turnover of DDX3X by the UPS. Importantly, the increased turnover rate of GlcNAc-deficient DDX3X^Ser584Ala^ was sufficient to impair S-phase entry. Indeed, the observed accumulation of DDX3X^Ser584Ala^ cells in G1 phase, but not S-phase, paired with the reduced population of G2/M phase cells points to delayed transitioning through the G1/S checkpoint following loss of DDX3X Ser584 O-GlcNAcylation, as opposed to delayed mitotic progression through M phase. Supporting this is the observation that cyclin E1 levels are downregulated in DDX3X^Ser584Ala^ cells, consistent with the previously documented role of DDX3X in promoting cyclin E1 mRNA translation, and our ability to rescue the accumulation of cells in G1 following DDX3X knock-down via cyclin E1 over-expression. Although mild mitotic stress can result in accumulation of cells in a quiescent G0 state, given that cyclin E functions in late G1 phase and over-expression rescues the G1 accumulation defect observed in *DDX3X* knock-down cells, our data support a pathway where DDX3X O-GlcNAcylation promotes S-phase entry rather than resulting in increased mitotic stress in M phase. Given the essential role cyclin E1 plays in CDK2 activation, DDX3X O-GlcNAcylation may contribute to CDK2 activation and downstream gene expression networks in late G1, thus explaining the S-phase entry defect.

Previously, chemical inhibition of OGT using acetyl-5S-GlcNAc in MCF-7 breast cancer cells was reported to cause a reduction in the percentage of S-phase cells without affecting DNA replication rate, indicative of impaired G1/S phase transitioning as opposed to prolonged S-phase duration, although the authors were unable to determine which O-GlcNAc signalling nodes were dysregulated to convey this effect [28]. Our data here indicate that DDX3X contribute to the stalled G1/S transition observed in this previous study. It is, however, likely that OGT inhibition additionally affects S-phase entry via other mechanisms unrelated to DDX3X, with DDX3X acting as one of many O-GlcNAc-dependent regulatory mechanisms that have yet to be identified. Relevant to this point is the prior observation that global O-GlcNAc levels decrease as cells enter S-phase [51], whereas O-GlcNAcylation of DDX3X promotes S-phase entry, indicating O-GlcNAc does not solely act as a promoter or repressor of S-phase entry. Indeed, other DEAD box RNA helicases, replication licensing factors, and cytoskeletal proteins are dynamically O-GlcNAcylated in late G1, although how O-GlcNAc modulates the activity of these proteins to regulate the G1/S transition is unclear [51]. To our knowledge, this study reports the first identification of a single O-GlcNAcylation event that regulates the G1/S transition, emphasizing the paucity of information on how O-GlcNAc regulates this critical point of the cell cycle and the need to further study this poorly understood area of biology.

DDX3X missense, frameshift and nonsense variants cause an ID syndrome that presents with autistic spectrum behaviours, delayed language acquisition, eye malformation and microcephaly [33,38]. In mouse models of DDX3X ID, the microcephaly observed in female DDX3X loss-of-function mice stemmed from reduced proliferation of neuronal progenitors during embryonic corticogenesis [57]. Interestingly, missense variants in OGT also give rise to an intellectual disability syndrome called the O-GlcNAc Transferase Congenital Disorder of Glycosylation (OGT-CDG) [58]. OGT-CDG is clinically heterogeneous, with different missense variants presenting with variable peripheral dysmorphias, muscle hypotonia, and neuroanatomical abnormalities including microcephaly [11,13–15,58]. Due to the plethora of processes regulated by O-GlcNAcylation, it is unclear which O-GlcNAc-dependent processes are dysregulated to cause microcephaly in OGT-CDG. Our results here suggest that hypo-O-GlcNAcylation of DDX3X could be a candidate conveyor of the microcephaly observed in OGT-CDG patients. With a recently reported mouse model of a catalytically deficient OGT-CDG variant [59], future work investigating the possible mechanistic link between DDX3X O-GlcNAcylation and microcephaly may yield insights into the aetiology of this poorly understood disease.

## 4. Material and methods

### 4.1. Analysis of prefrontal cortex microarray data

To investigate temporal *OGT* and *OGA* co-expression in the human prefrontal cortex as a strategy to identify candidate genes involved in the same cellular pathways, the BrainCloud dataset containing human post-mortem gene expression data from brain tissue was used [46]. The BrainCloud dataset was produced from transcriptomic analysis of RNA isolated from the dorsolateral prefrontal cortex Brodmann areas 46/9 grey matter tissue from foetal ages through ageing in 267 individuals with no severe neuropathological, neurological or neuropsychiatric diagnoses, across the human lifespan. Transcriptomic data were downloaded from the GEO repository (GSE30272) together with the annotation file and the demographic data. The different probes targeting the same gene were aggregated to their mean value, resulting in 17 160 genes being included in the subsequent analyses. All analyses were performed using RStudio (R v4.3.0). The correlation (*r*) was calculated for *OGT* and *OGA* and all other genes, respectively, using the cor()-function from the base R package stats (v3.6.2). Afterwards, gene lists of correlation and anti-correlation were extracted, including only genes with a *r* ≥ 0.4 (or *r* ≤ −0.4) and *p* < 0.05, Student’s asymptotic test. To identify which of the genes were specifically involved in cell cycle-related processes, the gene lists were submitted to the Enrichr online resource to perform GO enrichment analysis for biological processes. Enriched terms were only considered significant if *p* < 0.05 after Benjamini–Hochberg correction for multiple comparisons.

### 4.2. Cloning of DDX3X and OGT constructs for HEK293T transfection and *E. coli* expression

A PCR product containing the Human ORF for DDX3X was obtained directly from RNA using the Primescript High Fidelity RT-PCR kit from Takara. The PCR primers introduced *Bam*HI and *Not*I sites at the 5′ and 3′ ends, respectively. The fragment was cloned into pCMV-FLAG as a *Bam*HI-*Not*I restriction fragment. The insert was confirmed by DNA sequencing. Site-directed mutations at S584A, S584C, S588A and the double mutation S584A;S588A were introduced using site-directed mutagenesis based on the Quickchange mutagenesis kit, but KOD polymerase was used instead of Pfu. The sequence was verified again by sequencing.

To confer siRNA immunity on the DDX3X construct a geneblock was designed (from IDT—Integrated DNA Technologies) containing silent mutations to alter the sequences recognized by existing siRNAs flanked by complementary sequences. This sequence (shown below) was incorporated into the existing construct by restrictionless cloning based on [60]. The sequence was confirmed by DNA sequencing. Silent mutations introduced are shown below, with nucleotides in bold highlighting substituted nucleotides: Wild-type DDX3X sequence: 5′-CTATATTCCTCCTCATTTA-3′ SiRNA resistant DDX3X sequence: 5′-GTACATCCCACCACACCTG-3′. A P2A site and the ORF for superfolder (sf) GFP were obtained by PCR from a pre-existing mammalian co-expression vector by PCR. This was fused to the C-terminus of the existing human DDX3X expression vector by restrictionless cloning. This results in the separate expression of superfolder (sf) GFP in cells transfected with the plasmid in addition to DDX3X. DDX3X (132-607) was ordered as a codon-optimized gene block from Integrated DNA Technologies including an N-terminal *Bam*HI site, a C-terminal stop codon and a *Not*I site. This fragment was digested and cloned into a *Bam*HI-*Not*I digested and dephosphorylated vector pHEX6P1 (this proprietary vector is based on pGEX6P1 with a 6xHis tag in place of the GST tag). The full-length ORF for human OGT and OGA were cloned into pCMV-FLAG previously as PCR products generated from cDNA. A pre-existing mutant of OGT K842M was sub-cloned from pCMV-FLAG to pCMVHA to generate a HA-tagged inactive OGT protein.

### 4.3. HEK293T cell culture and transfection

HEK293T cells were cultured in DMEM/F12 medium, supplemented with 10% (v/v) FBS, 1 X GlutaMax, and 5 mL of 5000 U/mL penicillin/streptomycin. For transfection of CMV-vectors encoding FLAG-tagged constructs, the lipofectamine 3000 transfection kit (ThermoFisher) was used. Briefly, HEK293T cells were seeded at 30% confluency 16 h before transfection, and a 1 : 2 (w/v) ratio of plasmid to lipofectamine was used in all cases, following the manufacturer’s instructions. Transfections were carried out for 48 h, except for transfections with SiLuciferase, SiDDX3X, SIR WT or S584A, for which 50 000 cells were transfected and incubated for 72 h.

### 4.4. Cycloheximide chase assay

HEK293T cells were transfected with FLAG-DDX3X^WT^ or FLAG-DDX3X^Ser584Ala^ as described above. After 48 h, cycloheximide chase was initiated by the addition of 100 μg ml^−1^ cycloheximide for the indicated timepoints, after which cells were lysed and analysed by Western blotting.

### 4.5. Tandem metabolic labelling/SPAAC PEGylation

For investigating endogenous DDX3X O-GlcNAcylation stoichiometry, HEK293T cells were seeded at ~50% confluence, followed by addition of 200 μM Ac_4_GalNAz or an equal volume of DMSO (vehicle only control) for 16 h. The following day, cells were lysed in RIPA buffer and soluble protein extracted by centrifugation at 13 400 rpm in an Eppendorf mini-spin centrifuge. 200 μg protein was alkylated using iodoacetamide (22.5 mM final concentration; RT, 30 min, in the dark). After alkylation, proteins were chloroform/methanol precipitated by sequential addition of 600 μL MeOH, 150 μl chloroform, and 400 μl water, followed by centrifugation (13 400 × *g*, 5 min). The upper, aqueous, layer was removed, followed by addition of 450 μl MeOH and centrifugation (13 400 × *g*, 5 min) to pellet precipitated protein. Precipitated protein was resuspended in 90 μl 25 mM Tris-HCl, pH 8.0, 1% (w/v) SDS, and 10 μl of 10 mM DBCO-mPEG (5 kDa) added to initiate the SPAAC reaction. SPAAC reactions were incubated at 98°C for 5 min, followed by chloroform/ methanol precipitation and boiling of the precipitated protein in 45 μl 1 × LDS for SDS-PAGE/Western blotting. For investigating O-GlcNAcylation stoichiometry, HEK293T cells were transfected with FLAG-tagged DDX3X constructs for 48 h as described above. 32 h into the transfection, Ac_4_GalNAz was added to cell media for 16 h, and lysates were PEGylated as described above.

### 4.6. Recombinant protein expression and purification from *E. coli*

A 50 μl aliquot of NiCo21(DE3) *E. coli* was transformed with 1 μL HEXP1 plasmid encoding either His6-DDX3X^WT^ or His6-DDX3X^S584A^. Subsequently, 6 l of LB media (+100 μg ml^−1^ ampicillin) was inoculated with 10 ml/l of turbid starter culture derived from overnight culture of a single colony in 100 ml LB media (+100 μg ml^−1^ ampicillin). Cultures were grown to an OD600 of 0.6 at 37°C (125 rpm), after which expression was induced with 250 μM IPTG. Induced cultures were left overnight at 37°C (125 rpm). The following day, cultures were spun-down (4000 × *g*, 4°C, 40 min) and bacterial pellets resuspended in a minimal volume of lysis buffer (20 mM HEPES, pH 7.5, 250 mM NaCl, 30 mM imidazole, 0.5 mM TCEP, 1 mM benzamidine, 0.2 mM PMSF, 5 μM leupeptin, ~0.1 mg ml^−1^ lysozyme and ~0.1 mg ml^−1^ porcine DNase) and left overnight in a −80°C freezer. The following day, frozen pellets were thawed at room temperature and lysed via French press. Lysates were spun-down (50 000 × *g*, 4°C, 1 h) and immediately applied to 10 ml pre-washed Ni^2+^- NTA agarose, followed by incubation for 2 h at 4°C with rotation. Beads were washed 2 × with lysis buffer, 1 × with lysis buffer containing 1 M NaCl to remove bound nucleic acids, and 7 × more with lysis buffer. His6-DDX3X was eluted with 200 mM imidazole. Eluted protein was supplemented with 150 μl of 4 mg ml^−1^ PreScission protease for in-solution tag cleavage and simultaneously dialysed with imidazole-free lysis buffer overnight. After negative pulldown with an equal volume of fresh Ni^2+^-NTA beads, the eluent was concentrated to 5 ml and loaded on a HiLoad 16/600 Superdex 200 size exclusion column with imidazole-free lysis buffer as the mobile phase. Fractions containing protein at the expected Mw were pooled, buffer exchanged into 20 mM HEPES, pH 7.5, 500 mM NaCl, 10% (v/v) glycerol, 0.5 mM TCEP and concentrated to ~5 mg ml^−1^ prior to flash freezing.

### 4.7. Differential scanning fluorimetry

Purified DDX3X^WT^ (132-607) and DDX3X^S584A^ were diluted to 1 mg ml^−1^, and 125 μ of 1 mg ml^−1^ DDX3X (residues 132-607) spiked with 1 μl 5000 X SYPRO orange dye. In total, 25 μl of SYPRO orange/DDX3X mix was aliquoted across 6 wells of a clear PCR plate. A CFX connect real-time system was used to measure SYPRO orange fluorescence. After holding at 20°C for 5 s, SYPRO orange fluorescence was measured from 20°C to 95 °C over 30 min (2.5°C min^−1^). T_m_ values were calculated as described in [61].

### 4.8. Reverse transcription quantitative PCR (RT-qPCR)

In total, 40% confluent HEK293T cells were transfected with FLAG-empty vector or FLAG-OGT^WT^ for 48 h, after which mRNA was extracted using the RNeasy mini-kit (QIAGEN), following manufacturer’s instructions. cDNA synthesis was carried out using 1 μg of mRNA and the qScript cDNA synthesis kit (Quantabio), following manufacturer’s instructions. Reactions consisted of 10 μl PerfeCta SYBR Green FastMix (2 X stock) with 0.3 μl of 10 μM forward and reverse primer each, 4.4 μL milliQ water, and 5 μL 10 ng μl^−1^ cDNA. RT-qPCR amplification curves and C_q_ values were determined using a CFX connect real-time system (cycling conditions: 30 s 95°C, 10 s 95°C, 30 s 60°C; 40 cycles). Primers used for qPCR analysis are listed in electronic supplementary material, table S1.

### 4.9. SiRNA-rescue of cellular proliferation

HEK293T cells were plated at a density of 1 × 10^5^ cells per well in a 12-well plate and left overnight. Cells were subsequently transfected with 1 μg siRNA against DDX3X (siDDX3X) or Luciferase (siLuciferase), and 0.5 μg plasmid encoding siRNA-resistant FLAG-DDX3X^WT^ or FLAG-DDX3X^S584A^. Transfected cells were left for 3 days, after which media was aspirated, followed by 3 × PBS washes to remove dead cells. Cells were subsequently resuspended in 100 μl trypsin, followed by centrifugation at 300 × *g* (5 min). Trypsin was aspirated, and cell pellets were resuspended in 10% (v/v) FBS in PBS, followed by trituration to achieve a homogeneous single-cell suspension. After live cell counting using a LUNA-IITM automated cell counter, cells were spun-down again (300 × *g*, 5 min) prior to resuspension in RIPA lysis buffer.

### 4.10. Cell cycle analysis

SiRNA rescue of DDX3X was performed as described above. After siRNA rescue, trypsinized cells were centrifuged at 300 × *g* for 3 min, followed by removal of the supernatant. The cells were then resuspended in 1 ml of PBS with 1% (w/v) FBS. Centrifugation was then repeated at 300 × *g* for 3 min, and the resulting pellet was resuspended in 0.5 ml of PBS with 1% (w/v) FBS. To fix the cells, 4.5 ml of 70% ethanol at −20°C was added dropwise during gentle vortexing to maintain a homogeneous suspension. The cell suspension was then incubated at 4°C for 30 min. Following this, the suspension was centrifuged at 300 × *g* for 3 min, after which the supernatant was aspirated, and cells were rehydrated by the addition of 3 ml PBS supplemented with 1% (w/v) FBS for 2 min. After a further centrifugation at 300 × *g* for 3 min, the supernatant was removed, and the cells were resuspended in 50 µl of PBS containing 1% (w/v) FBS and 100 µg  ml^−1^ RNase A for 30 min at room temperature. Subsequently, PI was added to a final concentration of 1 µg  ml^−1^, followed by incubation in the dark at 4°C for at least 30 min, preparing the cells for flow cytometry analysis. The cell suspensions were analysed using a NovoCyte 3000 flow cytometer (Agilent, Santa Clara, CA) via a 488 nm laser. The 488/10 detector was used for forward side scatter (FSC) and side scatter (SSC), and the 615/20 detector for PI detection. Data acquisition and analysis were performed using NovoExpress software (version 1.6.2, Agilent, Santa Clara, CA). During data acquisition, multiple gating strategies were employed to isolate single, PI-stained cells for cell cycle analysis. Initially, cells were gated based on size and granularity using FSC area (FSC-A) and SSC area (SSC-A). These gated cells were then analysed by comparing FSC height (FSC-H) to FSC-A to identify single cells. The single cells were further plotted on a PI area (PI-A) versus FSC-A graph to select PI-stained cells. Finally, these gated cells were subjected to cell cycle analysis using the built-in function of the software, employing the Watson Pragmatic model for data fitting.

### 4.11. Annexin V and propidium iodide staining of HEK293T cells

HEK293T cells were transfected with siRNA targeting *DDX3X* or siRNA against firefly luciferase for 48 h, as described above. A positive control was included using 2 µM Staurosporine treatment for 6 h, alongside a DMSO-treated condition. Following treatment, cells were washed with ice-cold PBS, centrifuged at 300 × *g* for 3 min and resuspended in annexin-binding buffer (10 mM HEPES, 140 mM NaCl, 2.5 mM CaCl₂, pH 7.4). The cell density was adjusted to approximately 1 × 10⁶ cells per ml in annexin-binding buffer after cell counting. A 100 μL aliquot of each suspension was transferred to a new tube, and 5 μl of Annexin V conjugated to Alexa Fluor 647 (Thermo Fisher) and 1 μl of 10 mg ml^−1^ PI was added. The samples were incubated at room temperature for 15 min. Subsequently, 400 μl of annexin-binding buffer containing 1 mg ml^−1^ PI was added to each sample, mixed gently, and the samples were placed on ice for immediate analysis by flow cytometry.

Flow cytometry was performed using the NovoCyte 3000 flow cytometer, employing the same gating strategy and filter channels as previously described. For Annexin V—Alexa Fluor 647 detection, the 640 nm laser and 675/30 nm detector were used. Singlet cells were gated, and the Annexin V versus PI plots were analysed to categorize cells into live, early apoptotic, late apoptotic or necrotic populations.

### 4.12. MTT assay

To identify the linear range in which the number of viable cells is directly proportional to formazan absorbance at 560 nm, a standard curve was generated by plating 2.5 × 10^4^ to 2.5 × 10^5^ HEK293T cells in a 12-well plate. After overnight incubation, cell culture media was removed and 1 ml of 0.5 mg ml^−1^ thiazolyl blue tetrazolium bromide, dissolved in media, was added to each well. Cells were incubated for 4 h prior to removal of the cell culture medium and addition of 100 μl DMSO for 5 min to solubilize formazan crystals. DMSO-solubilized formazan was transferred to a 96-well plate and absorbance at 560 nm measured using a SpectraMax iD5. To ensure cell viability counts were within the linear range of detection, siRNA rescue experiments were performed using 1 × 10^5^ cells. After 72 h, the MTT assay was repeated as described above, with the additional step of diluting the DMSO-solubilized formazan crystals 1 : 16 in DMSO. In total, 100 μl of the diluted formazan crystals was transferred to a 96-well plate and absorbance measured at 560 nm as described above.

### 4.13. Western blotting

40 μg of PEGylated glycoprotein or 20 μg of cell lysate were run on a 4%–12% NuPAGE Bis-Tris gel for 2 h (120 V). Proteins were transferred onto nitrocellulose membrane using an Invitrogen semi-dry transfer system for 1 h (20 V). After blocking in 5% (w/v) bovine serum albumin dissolved in TBS (+0.1% (v/v) Tween-20) for 30 min (RT) membranes were incubated with the indicated antibodies for 1−2 h at RT, followed by 3 × 5 min TBS-T washes. Membranes were then incubated with the corresponding secondary antibody for 1 h (RT), prior to imaging using a Li-COR Odyssey.

### 4.14. Statistics

Western blot band signals and Coomassie blue staining intensities were integrated using ImageStudioLite (v 5.2.5) and statistical analyses carried out in GraphPad Prism (v 9.0). All statistical analyses pertaining to the BrainCloud atlas were performed in RStudio (see above for details). Sigmoidal, monophasic, curves were extracted from DSF data and analysed in GraphPad Prism (v 9.0). Throughout this manuscript, ns: not significant; *p < 0.05; **p < 0.01; ***p < 0.001; **** *p* < 0.0001.

## Data Availability

Supplementary material is available online [62].
